# Draft Genome Sequence of the Multidrug-Resistant *Escherichia coli* Strain LR09, Isolated from a Wastewater Treatment Plant

**DOI:** 10.1128/genomeA.00272-14

**Published:** 2014-04-03

**Authors:** Susan R. Leonard, David W. Lacher, Christopher A. Elkins, Carina M. Jung

**Affiliations:** a Division of Molecular Biology, Center for Food Safety and Applied Nutrition, U.S. Food and Drug Administration, Laurel, Maryland, USA; b U.S. Army Engineer Research and Development Center, Environmental Laboratory, Vicksburg, Mississippi, USA

## Abstract

We report the draft genome sequence of *Escherichia coli* O1:H6 strain LR09, which was isolated from a wastewater treatment plant and displays high resistance to five fluoroquinolone antimicrobials. The assembled data determine that the strain clusters with *E. coli* phylogroup F and harbors a plasmid conferring resistance to a broad spectrum of antibiotics.

## GENOME ANNOUNCEMENT

Wastewater treatment plants provide an opportunity for the dissemination of mobile antimicrobial resistance determinants as bacteria previously exposed to antibiotics mix with naive strains (1). However, these multidrug-resistant bacteria have been otherwise poorly characterized. Here we report the draft genome sequence of *Escherichia coli* LR09, isolated in August 2006 from the Little Rock Wastewater Utility treatment plant in Little Rock, AR (2). This strain was isolated using media containing 200 ng µl^-1^ norfloxacin and was shown to be highly resistant to all fluoroquinolones tested (2), as well as to gentamicin, streptomycin, and ampicillin (all above 200 ng µl^-1^).

Genomic DNA was extracted from an overnight culture using the DNeasy blood and tissue kit (Qiagen). Sequencing libraries were prepared with both the TruSeq DNA sample prep kit (Illumina) and the Nextera mate pair sample prep kit (Illumina) and sequenced on the Illumina MiSeq platform, generating 12,384,122 paired-end 300-bp reads and 9,355,464 mate pair 250-bp reads, respectively. The draft genome sequence was assembled *de novo* with CLC Genomics Workbench version 6.5.1 (CLC bio), producing 124 contigs resulting in a total genome size of 5,338,463 bp and an *N*_50_ of 347,262 bp. Plasmid DNA was isolated using the Large-Construct kit (Qiagen) and sequenced to determine which contigs comprised plasmid DNA; however, closure of the plasmid sequences was not performed. The draft genome sequence was annotated using RAST (3) and predicted to contain 5,183 coding sequences and 88 tRNAs.

Phylogenetic analysis utilizing backbone single nucleotide polymorphisms (SNPs) from whole-genome sequences revealed that LR09 clusters with *E. coli* phylogroup F (4). Molecular serotyping using the *wzx*, *wzy*, and *fliC* loci indicated that LR09 is O1:H6. BLAST results were negative for molecular markers of known *E. coli* pathotypes, including toxins and toxin genes (*stx*_1_, *stx*_*2*_, *ehxA*, *hlyA*, LT, and ST) and genes for adhesins (*eae*, *bfpA*, *aggR*, and *pap*). The genome contains two small plasmids, a 1,460-bp plasmid with 99% sequence identity to *E. coli* strain 1/21 plasmid pKST21 (GenBank accession no. JF436966.1) and a 7-kb plasmid encoding a colicin and a DNA restriction and modification system. In addition, LR09 possesses a 92-kb plasmid homologous to p12579_1 from *E. coli* O55:H7 strain RM12579 (GenBank accession no. CP003110.1). This plasmid is a lysogenic bacteriophage that remains circularized and extrachromosomal (5). A fourth plasmid harbored in LR09 is a large (>163 kb) IncF virulence plasmid exhibiting homology to pRSB225 (GenBank accession no. JX127248.1) carried by an uncultured bacterium isolated from a wastewater treatment plant in Germany (6). Along with genes encoding putative virulence factors, this LR09 plasmid carries antibiotic resistance genes identified by ResFinder (7). These include genes for resistance to aminoglycosides [*strAB*, *aadA5*, and *aac*(*3*)*-IId*], beta-lactam (*bla*_TEM-1B_), macrolide [*mph*(A)], chloramphenicol (*catA1*), sulfonamide (*sul1*), tetracycline [*tet*(B)], and trimethoprim (*dfrA17*). In addition, the genome contains an antibiotic resistance cassette conferring resistance to beta-lactam (*bla*_OXA-1_), chloramphenicol (*catB3*), and aminoglycosides and fluoroquinolones [*aac*(*6*′)-*Ib-cr*], the latter gene of which was previously shown to be responsible for the acetylation of ciprofloxacin and norfloxacin in this isolate (2).

### Nucleotide sequence accession number.

The draft genome sequence of *E. coli* LR09 was deposited at DDBJ/EMBL/GenBank under accession no. JDVF00000000.

## References

[1] Schlüter A Szczepanowski R Pühler A Top EM 2007 Genomics of IncP-1 antibiotic resistance plasmids isolated from wastewater treatment plants provides evidence for a widely accessible drug resistance gene pool. FEMS Microbiol. Rev. 31:449–477. 10.1111/j.1574-6976.2007.00074.x 17553065

[2] Jung CM Heinze TM Strakosha R Elkins CA Sutherland JB 2009 Acetylation of fluoroquinolone antimicrobial agents by an *Escherichia coli* strain isolated from a municipal wastewater treatment plant. J. Appl. Microbiol. 106:564–571. 10.1111/j.1365-2672.2008.04026.x 19200322

[3] Aziz RK Bartels D Best AA DeJongh M Disz T Edwards RA Formsma K Gerdes S Glass EM Kubal M Meyer F Olsen GJ Olson R Osterman AL Overbeek RA McNeil LK Paarmann D Paczian T Parrello B Pusch GD Reich C Stevens R Vassieva O Vonstein V Wilke A Zagnitko O 2008 The RAST server: rapid annotations using subsystems technology. BMC Genomics 9:75. 10.1186/1471-2164-9-75 18261238 PMC2265698

[4] Jaureguy F Landraud L Passet V Diancourt L Frapy E Guigon G Carbonnelle E Lortholary O Clermont O Denamur E Picard B Nassif X Brisse S 2008 Phylogenetic and genomic diversity of human bacteremic *Escherichia coli* strains. BMC Genomics 9:560. 10.1186/1471-2164-9-560 19036134 PMC2639426

[5] Kyle JL Cummings CA Parker CT Quiñones B Vatta P Newton E Huynh S Swimley M Degoricija L Barker M Fontanoz S Nguyen K Patel R Fang R Tebbs R Petrauskene O Furtado M Mandrell RE 2012 *Escherichia coli* serotype O55:H7 diversity supports parallel acquisition of bacteriophage at Shiga toxin phage insertion sites during evolution of the O157:H7 lineage. J. Bacteriol. 194:1885–1896. 10.1128/JB.00120-12 22328665 PMC3318487

[6] Wibberg D Szczepanowski R Eikmeyer F Pühler A Schlüter A 2013 The IncF plasmid pRSB225 isolated from a municipal wastewater treatment plant’s on-site preflooder combining antibiotic resistance and putative virulence functions is highly related to virulence plasmids identified in pathogenic *E. coli* isolates. Plasmid 69:127–137. 10.1016/j.plasmid.2012.11.001 23212116

[7] Zankari E Hasman H Cosentino S Vestergaard M Rasmussen S Lund O Aarestrup FM Larsen MV 2012 Identification of acquired antimicrobial resistance genes. J. Antimicrob. Chemother. 67:2640–2644. 10.1093/jac/dks261 22782487 PMC3468078

